# Efficacy of oral midazolam for minimal and moderate sedation in pediatric patients: A systematic review

**DOI:** 10.1111/pan.13747

**Published:** 2019-10-14

**Authors:** Maria A. Manso, Catherine Guittet, François Vandenhende, Luc‐André Granier

**Affiliations:** ^1^ Clinical Affairs Advicenne, Nîmes France; ^2^ Statistics ClinBay Genappe Belgium

**Keywords:** benzodiazepines, clinical efficacy, hypnotics and sedatives, pediatrics, preanesthetic medication, randomized trials

## Abstract

One of the most widely used options for minimal/moderate sedation in pediatric patients is oral midazolam, as it presents an alternative to less well‐accepted routes of administration (*eg,* intravenous or intranasal) of this well‐known efficacious and well‐tolerated short‐acting benzodiazepine. A systematic review of the literature was conducted in order to identify clinical studies evaluating the effectiveness of oral midazolam for sedation in pediatric patients in the context of premedication before anesthesia or during diagnostic/treatment procedures. The percentage of responders (response rate) after single administration of oral midazolam was evaluated and compared versus placebo in a subset of placebo‐controlled studies. The range of oral midazolam doses providing effective sedation in the different pediatric age subsets was analyzed in order to assess optimum dosing strategies. A total of 25 pediatric clinical studies, utilizing a variety of measures of sedation effectiveness, were selected. These studies included a total of 1472 patients (aged 4 months‐18 years) treated with midazolam (0.25‐1.5 mg/kg) and 138 patients treated with placebo. The response rates [95% confidence interval] with oral midazolam ranged from 36.7% [21.6%, 54.9%] to 97.8% [86.1%, 99.7%], while with placebo response rates ranged from 4.0% [0.6%, 23.5%] to 41.0% [29.4%, 53.6%]. When considering the 4 placebo‐controlled studies, the odds ratios [95% confidence interval] for the comparison of midazolam vs. placebo ranged from 13.4 [5.0, 36.0] to 25.9 [6.7, 100.6]. The analysis of subgroups by context of sedation showed response rates [95% confidence interval] with oral midazolam ranging from 36.7% [21.6%, 54.9%] to 97.0% [94.8%, 98.3%] for anesthetic premedication and from 56.1% [43.1%, 68.4] to 97.8% [86.1%, 99.7%] for medical procedures. The efficacy of midazolam for pediatric minimal/moderate sedation from a dose of 0.25 mg/kg and above was demonstrated. The probability of occurrence of adverse events and over‐sedation increases with increasing doses.

## 1 INTRODUCTION

It is estimated that more than 50% of children could benefit from minimal to moderate sedation during perioperative or procedural periods to treat or prevent behavioral stress and anxiety, caused by separation from their families, the presence of an unfamiliar environment, or fear of pain.^1^, ^2^, ^3^, ^4^

The objective of minimal/moderate sedation is to enable the accomplishment of a scheduled intervention with a child who is calm, in order to prevent psychological distress prior to or during the intervention, to avoid poor compliance or cancelation, and any potential negative impact on postoperative recovery or other possible long‐term psychological consequences.^4^, ^5^, ^6^

Midazolam has a very long track record of use for minimal and moderate sedation and remains the most commonly used oral sedative for anxiolysis in children. A recent Cochrane review evaluated midazolam for sedation before procedures and discussed data about the effectiveness of midazolam in adults and pediatric patients (by any route) in comparison with other medications using different outcome measures.^7^ Among the drugs used for moderate sedation, oral midazolam offers the advantage of being an efficacious, short‐acting benzodiazepine, with anxiolytic, sedative, and hypnotic properties, with a favorable benefit/risk ratio.^8^ Since the late 1980s, a number of clinical trials have been published evaluating the efficacy of oral midazolam for sedation in children; hence, a large volume of information relating to oral midazolam as a sedative in pediatric patients is available. There is, however, a broad range of oral doses used in pediatric patients for minimal/moderate sedation, and the optimum dosing in different contexts of sedation remains unclear.

The major aims of the present review were to summarize and analyze the available literature data related to minimal/moderate sedation with oral midazolam in pediatric patients, both prior to anesthesia and during minor procedures, and to evaluate oral midazolam doses providing effective minimal or moderate sedation in different pediatric age subsets and contexts of sedation.

## 2 RESEARCH STRATEGY

### 2.1 Literature search

A systematic review of the literature was conducted in order to identify clinical studies evaluating the use of oral midazolam in pediatric patients in the context either of premedication before anesthesia for surgical procedures or during diagnostic or treatment procedures. Articles were identified from electronic resources including PubMed and ScienceDirect (*eg,* MEDLINE search ("midazolam"[MeSH Terms] OR "midazolam"[All Fields]) AND “sedation”[All Fields] AND ("child"[MeSH Terms] OR "child"[All Fields] OR "children"[All Fields]) AND ("pediatrics"[MeSH Terms] OR "pediatrics"[All Fields] OR "pediatric"[All Fields])) and by manual searches from further evaluation of key review articles and bibliographies of articles. All studies published or in press between January 1988 and March 2016, and written in English, were considered.

### 2.2 Study selection and outcomes

Only randomized studies that assessed the efficacy of midazolam as a sole medication using evaluation scales related to minimal/moderate sedation, and where the effectiveness of oral midazolam for sedation in terms of number of responders with respect to the number of treated children (generally within 30‐45 minutes postadministration, and up to 1 hour) was reported, were considered. All studies evaluating moderate sedation using discrete sedation scales were selected.

The outcome used for comparison purposes was the proportion of patients considered adequately sedated for the specific intervention (response rate), as detailed in each study. Studies that used placebo, other drugs, different oral midazolam doses, different oral midazolam preparations, or other routes of administration as comparators were acceptable. Information about comparators used in the different studies was extracted. Observations regarding vital signs and reported adverse events were summarized.

### 2.3 Classification of literature data and statistical methods

**Figure 1:**
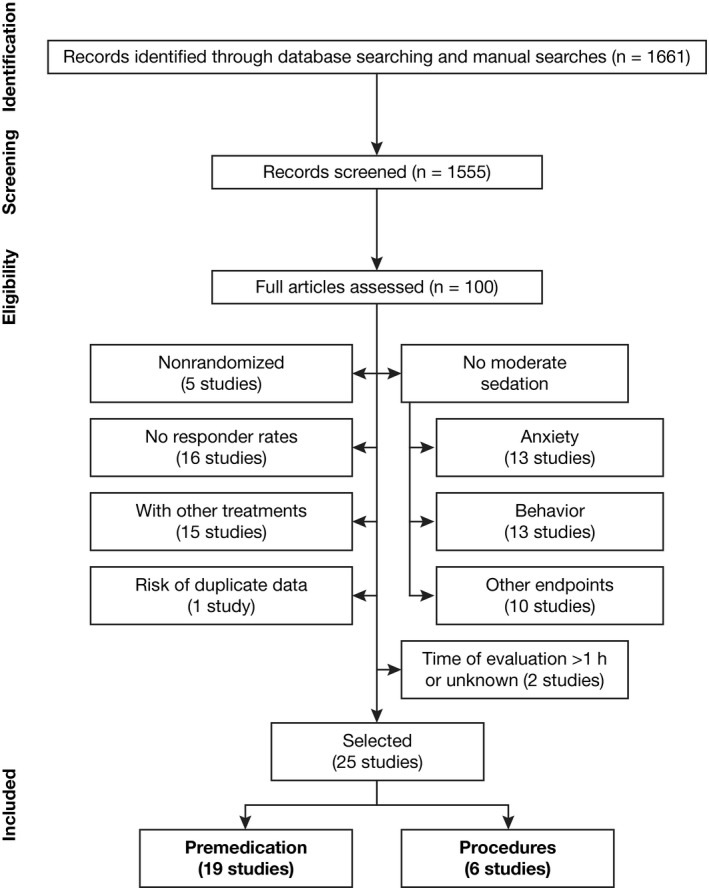
Flow diagram of study selection process

As shown in Figure 1, the initial search strategy identified 1661 records. A total of 100 articles were assessed for eligibility by full‐text assessment by at least 2 reviewers. The 25 articles selected for inclusion in the review involve a total of 1610 patients, aged from 4 months to 18 years old; 1472 patients were treated with a single oral midazolam dose between 0.25 and 1.5 mg/kg, with a maximum dose of 20 mg used most frequently (40 mg in one of the studies),^9^ and 138 patients were treated with placebo (Table 1).

**Table 1:** Number of selected studies (number of patients) according to different classification criteria

	Oral midazolam	Placebo
Overall	25 (1472)	4 (138)
Blinding		
Open	5 (310)	
Blind observer	4 (161)	
Double‐blind	16 (1001)	4 (138)
Context of sedation		
Anesthetic premedication	19 (1246)	3 (106)
Medical procedures	6 (226)	1 (32)
ASA status		
ASA I‐II	19 (710)	4 (138)
ASA I‐III	2 (482)	
ASA ≥ II	2 (199)	
ASA UNK	2 (81)	
Dose		
0.25 mg/kg^a^	5 (236)	
0.5 mg/kg	22 (811)	
0.75 mg/kg	3 (55)	
1.0 mg/kg	5 (234)	
1.5 mg/kg	1 (136)	
Form		
Preparation	21 (810)	
Oral syrup	5 (614)	
IV form	1 (48)	
Type of success criterion		
A	12 (1020)	3 (113)
B	11 (377)	
C	2 (75)	1 (25)

Abbreviations: A, nonrestrictive success criterion; B, restrictive success criterion; C, criterion based on the OAA/S score; IV, intravenous; UNK, unknown.

a Includes 0.25‐0.3 mg/kg doses.

A summary description of the individual clinical efficacy studies for both contexts of sedation, including efficacy results per treatment (midazolam and placebo) and dose, is shown in Table 2. The data from the literature were tabulated, indicating the number of subjects, number of responders, and response rates per treatment. Whenever sedation levels only were indicated or different time points were presented, the sedation success criterion was defined as indicated in Table 2.

**Table 2:** Summary description of the individual clinical efficacy studies using oral midazolam for anesthetic premedication and procedural sedation in pediatric patients

Study	Blinding	Intervention	Evaluation scale (success criterion, evaluation time point)	Type of success criterion	Age range	ASA status	Treatment group (formulation type)	Dose (mg/kg)	n/N	Other comparators	Safety with midazolam
Anesthetic premedication											
Alderson 1994^27^	Double‐blind	Ambulatory dental surgery	Sedation 4‐point scale (3 = drowsy at induction, 21.8 ± 7.0 min postadministration)^a^	B	1‐6 y	ASA I‐II	Oral midazolam (extemporaneous preparation, mixture of IV form with syrup)	0.5	19/20	Oral ketamine 5 mg/kg	Postoperative pain in 4 children, postoperative vomiting in one child, nightmares after surgery in one child
Brosius 2002^10^	Double‐blind	Superficial surgical procedure	OAA/S sedation 20‐point scale (score ≤ 17, 29.0 ± 4.8 min, range 23‐40 min, postadministration)	C	10‐18 y	ASA I‐II	Oral midazolam (extemporaneous preparation, mixture of IV form with syrup)	0.3	10/25	No other comparators	Not reported
							Placebo	‐	1/25		
Brosius 2003^13^	Double‐blind	Surgical procedure	OAA/S sedation 20‐point scale (score ≤ 17, 30 min postadministration)	C	2‐10 y	ASA I‐II	Oral midazolam (extemporaneous preparation, mixture of IV form with syrup)	0.5	23/25	No other comparators	Deep sedation in 10% of the patients
							Oral midazolam (oral syrup, Versed^®^)	0.5	16/25		
Coté 2002^18^	Double‐blind	Surgical procedure	Sedation 5‐point scale (3 = relaxed, 4 = drowsy or 5 = asleep, within 30 min postadministration)	A	6 mo‐1 y	ASA I‐III	Oral midazolam (oral syrup, Versed^®^)	0.25	45/48	No other comparators	Before induction: nausea (2 cases at high dose), emesis (one case at each dose). One case of upper airway obstruction with initiation of N_2_O at high dose. Overall incidence of respiratory disorders increased with the dose
								0.5	47/49		
								1	50/50		
					2‐5 y	ASA I‐III	Oral midazolam (oral syrup, Versed^®^)	0.25	38/43		
								0.5	38/38		
								1	42/42		
					6‐16 y	ASA I‐III	Oral midazolam (oral syrup, Versed^®^)	0.25	40/41		
								0.5	44/45		
								1	41/41		
Damle et al 2008^20^	Double‐blind	Premedication for dental surgery under general anesthesia in noncooperative children	Sedation 4‐point scale (3 = drowsy, 4 = asleep, 30 min postadministration) a	B	2‐6 y	ASA I‐II	Oral midazolam (extemporaneous preparation, mixture with honey)	0.5	9/10	Oral ketamine 5 mg/kg	Vomiting in 10% of the children
Darlong 2004^28^	Blind observer	Elective ophthalmic surgery	Sedation 5‐point scale (3 = drowsy or 4 = asleep, 30 min after administration)	B	1‐9 y	ASA I‐II	Oral midazolam (extemporaneous preparation, mixture with honey)	0.5	16/24	Oral ketamine 6 mg/kg or combination of oral ketamine 3 mg/kg + oral midazolam 0.25 mg/kg	Postoperative nausea and vomiting (25% of the cases)
Darlong 2011^33^	Double‐blind	Ophthalmic surgery	Sedation 5‐point scale (3 = drowsy or 4 = asleep, 30 min after administration)	B	1‐10 y	ASA I‐II	Oral midazolam (extemporaneous preparation, mixture with honey)	0.5	12/29	Combinations of oral ketamine 3 mg/kg + oral midazolam 0.25 mg/kg or oral ketamine 6 mg/kg + oral midazolam 0.5 mg/kg	Postoperative nausea and vomiting (34.5% of the cases) and one case of irrelevant talking
Debnat 2003^26^	Open	Minor surgery	Sedation 5‐point scale (3 = drowsy or 2 = light sleep, 30 min postadministration) a	B	1‐10 y	ASA I‐II	Oral midazolam (extemporaneous preparation, mixture of IV form with sugar)	0.5	11/30	Oral ketamine 6 mg/kg	Preoperatively: euphoria (80%), hiccough (10%). Postoperatively: irritability (70%)
Funk 2000^29^	Double‐blind	Surgery of duration > 30 min	Sedation 4‐point scale (3 = drowsy or 4 = asleep, 20 min postadministration at transfer to the operating room)	B	2‐10 y	ASA I‐II	Oral midazolam (extemporaneous preparation, mixture with syrup)	0.5	22/38	Oral ketamine 6 mg/kg or Combination of oral ketamine 3 mg/kg + oral midazolam 0.5 mg/kg	Before induction: 1 vertigo, 3 psychedelic symptoms, 1 excitation, 6 salivation
Ghai 2005^24^	Double‐blind	Surgery	Sedation 4‐point scale (1 = asleep, 2 = drowsy or 3 = calm, 20 ± 5.6 min from administration to induction of anesthesia)	A	10 mo‐6 y	ASA I‐II	Oral midazolam (IV form, administered orally)	0.5	46/48	Combination of oral ketamine 2.5 mg/kg + oral midazolam 0.25 mg/kg	Nausea and vomiting in 3 patients
Kogan 2002^31^	Double‐blind	Minor surgery under general anesthesia	Sedation 3‐point scale (2 = drowsy or 3 = asleep, around 30 min postadministration)	B	1‐5 y	ASA I‐II	Oral midazolam (extemporaneous preparation, mixture with syrup)	0.5	23/29	Intranasal midazolam 0.3 mg/kg or rectal midazolam 0.5 mg/kg or sublingual midazolam 0.3 mg/kg	No complications observed
Levine 1993a^34^	Blind observer	Ambulatory surgery	Sedation 4‐point scale (2 = awake/calm or 3 = drowsy, 10‐30 min postadministration, at separation from parents)	A	1‐6 y	ASA I‐II	Oral midazolam (extemporaneous preparation, mixture of IV form with syrup)	0.5	27/30	Comparison of different times of separation from parents	Mean HR and systolic BP values increased at mask application compared with baseline
Levine 1993b^35^	Open	Cardiac surgery (congenital cardiac disease)	Sedation 4‐point scale (2 = awake/calm or 3 = drowsy, 30 min postadministration, at separation from parents) a	A	1‐6 y	ASA ≥ II	Oral midazolam (extemporaneous preparation, mixture with syrup)	0.75	11/15	Oral or rectal pentobarbitone 2 mg/kg + intramuscular morphine 0.2 mg/kg and atropine 0.02 mg/kg	No clinically significant changes in HR and SpO_2_
Liacouras 1998^15^	Double‐blind	IV line placement before endoscopy	Sedation 4‐point scale (alert/aware, drowsy/not sleeping and sleeping, pre‐IV line placement around 20 min postadministration)	A	2‐18 y	ASA I‐II	Oral midazolam (extemporaneous preparation, mixture of IV form with syrup)	0.5	56/62	No other comparators	Similar frequency of minor side effects with midazolam (26%) and with placebo (25%)
							Placebo	‐	25/61		
Marshall 2000^9^	Double‐blind	Surgery under general anesthesia or minor procedure	Sedation 5‐point scale (3 = drowsy, or 4/5 = asleep, within 30 min postadministration)	B	6 mo‐15 y	ASA I‐III	Oral midazolam (oral syrup, Versed^®^)	0.25	18/28	No other comparators	11 adverse events in 21% of the patients
								0.5	20/24		19 adverse events in 46% of the patients
								1	31/33		22 adverse events in 42% of the patients
Masue 2003^23^	Open	Cardiovascular surgery (congenital heart disease)	Sedation 5‐point scale (3 = calm, 4 = drowsy or 5 = asleep, 30 min postadministration)	A	4 mo‐2 y	ASA ≥ II	Oral midazolam (extemporaneous preparation, mixture of IV form with syrup)	0.5	7/20	No other comparators	Severe salivation and drop in SpO2 in 4 children. Upper airway compromise in 4% of the cases at high dose
								1	19/28		
								1.5	122/136		
McMillan 1992^16^	Double‐blind	Minor surgery	Sedation 4‐point scale (2 = calm or 3 = drowsy at time of separation from parents, 30 min postadministration) a	A	1‐6 y	ASA I‐II	Oral midazolam (extemporaneous preparation, mixture of IV form with syrup)	0.5	15/20	No other comparators	No AEs
								0.75	16/20		4 loss of balance, and head control, 1 blurred vision, 1 dysphonic reaction, 1 ataxia upon discharge
								1	13/20		5 loss of balance, and head control, 1 blurred vision, 1 dysphonic reaction, 1 ataxia upon discharge
							Placebo	‐	3/20		No AEs
Sheta and AlSarheed 2009^21^	Double‐blind	Premedication for dental surgery under general anesthesia in noncooperative children	Sedation 5‐point scale (2 = calm or 3 = drowsy, in the operating room before induction, premedication was 30 min before parental separation)	A	2‐6 y	ASA I‐II	Oral midazolam (extemporaneous preparation, mixture of IV form with juice)	0.5	13/20	No other comparators	1 and 4 cases of over‐sedation at 0.75 and 1.0 mg/kg, respectively. Delayed recovery at 1.0 mg/kg
								0.75	16/20		
								1	16/20		
Talon 2009^30^	Blind observer	Reconstructive surgery under general anesthesia	Sedation 4‐point Ramsey‐like scale (1 = sleepy or 2 = alert/calm on arrival in the operating room, administration 30‐45 min before surgery)	A	1‐18 y	UNK	Oral midazolam (oral syrup, Roxane Laboratory.)	0.5	41/50	Intranasal dexmedetomidine 2 µg/kg	No AEs observed
Procedural sedation											
Jain 2010^17^	Double‐blind	Venepuncture before CT imaging	Sedation 5‐point scale based on behavior (3 = calm or 4 = drowsy, 20‐30 min postadministration) a	A	1‐5 y	ASA I‐II	Oral midazolam (extemporaneous preparation, mixture with honey)	0.5	24/29	Combination of oral ketamine 1.0 mg/kg + oral midazolam 0.25 mg/kg	Not reported
							Placebo	‐	5/32		
Klein 2011^32^	Blind observer	Laceration repair	Sedation 5‐point scale (3 = drowsy or 4 = calm, during procedure 11‐68 min postdose, median 34 min)	A	6 mo‐7 y	ASA I‐II	Oral midazolam (oral syrup, Roxane Laboratory.)	0.5 (max. 15 mg)	32/57	Intranasal midazolam 0.3 mg/kg or buccal midazolam 0.3 mg/kg	1 deep sedation; 1 pre‐ and 1 postdischarge vomiting, 1 postdischarge nightmares
Shapira et al 2004^22^	Double‐blind	Dental treatment under N_2_O 50% in noncooperative children	Sedation 5‐point scale (2 = quiet>50% of the time, 1 = quiet>50% of the time, just before the procedure, 20‐30 min after administration)	A	1‐4 y	ASA I‐II	Oral midazolam (extemporaneous preparation, liquid)	0.5	23/28	Combination of hydroxyzine 3.7 mg/kg + midazolam 0.3 mg/kg	Vital signs remained stable
Silver et al 1994^19^	Double‐blind	Dental procedure in noncooperative children	Successful sedation (glazed look or delayed eye movements, lack of muscle coordination, slurred speech or sleep), onset of sedation 25‐30 min after administration.	B	3‐18 y	ASA UNK	Oral midazolam (extemporaneous preparation, IV form with grape‐flavored suspension)	0.3	12/16	No other comparators	No clinical signs of compromised respiratory rate. No postoperative complications
								0.5	9/15		
Wilson 2002^36^	Open	Dental procedure	Sedation Brietkopf and Buttner 4‐point scale for emotional status (3 = inactive or 4 = sleepy in 5‐65 min, median = 20 min) a	B	10‐16 y	ASA I‐II	Oral midazolam (extemporaneous preparation, mixture with syrup)	0.5	45/46	Nitrous oxide 30% in oxygen via a nasal mask	No major AEs reported. 1 paradoxical reaction (disinhibition)
Wilson 2006^37^	Open	Dental extraction under local anesthesia	Sedation Brietkopf and Buttner 4‐point scale for emotional status (3 = inactive or 4 = sleepy in 2‐30 min, mean = 15.9 min) a	B	5‐10 y	ASA I‐II	Oral midazolam (extemporaneous preparation, mixture with syrup)	0.3	32/35	Nitrous oxide 30% in oxygen via a nasal mask	Drowsiness and headache on returning home in 20% of the children

Abbreviations: A, nonrestrictive success criterion; AEs, adverse events; ASA, American Society of Anesthesiologists; B, restrictive success criterion; C, success criterion based on the OAA/S score; CT, computerized tomography; IV, intravenous; Max., maximum; Min., minimum; n, number of responders; N, number of subjects; OAA/S, Observer's Assessment of Alertness/Sedation; UNK, unknown.

a Success criteria defined a posteriori.

The response rates (95% CI) and odds ratios (OR, 95% CI) were calculated for the individual studies and depicted graphically. Statistical analyses were carried out using SAS v 9.4 under PC windows.

## 3 DISCUSSION

The relevant literature on the use of midazolam in minimal/moderate sedation for children was reviewed. Variability in the assessment of sedation was observed between studies. However, there was a clear superiority of midazolam when compared to placebo in the range of doses evaluated.

### 3.1 Evaluation of effectiveness

Sedation was measured using a variety of discrete sedation scales, with a sedation success criterion defined for each study. The scales used in the studies for the evaluation of sedation in the present review were mainly 3‐ to 5‐point scales, and two studies were identified that utilized the Observer's Assessment of Alertness/Sedation (OAA/S) scale. It was, however, not possible to find a group of studies with perfectly consistent and relevant success criteria. Also, it was noted that the sedation levels “drowsy” or “awake but calm” could have slightly different meanings depending on the studies and that there could be a level of overlap of terminology between studies. The difficulty in finding a homogeneous group of studies in terms of sedation outcomes, together with the low quality of evidence of most of the studies (study limitations, imprecision, and risk of bias), precluded a meta‐analysis with a direct comparison of the effectiveness of midazolam at various doses, despite the amount of available data.

Nevertheless, the objective of this review was to report the data collected on the experience with oral midazolam in producing effective minimal/moderate sedation, and, as such, the different assessment methods were described as part of the reporting of results. However, conclusions could only be drawn from the results of individual studies. The difficulty in comparing the results from the different studies highlights the need to use validated scales for the evaluation of sedation.

The review of the selected articles indicated that three types of studies could be distinguished, according to the type of success criterion: (a) those using nonrestrictive criteria (ie, including awake/calm children as responders); (b) those using more restrictive criteria (ie, requiring at least drowsiness for a successful response); and (c) those using a criterion based on a sedation threshold using a validated scale, the Observer's Assessment of Alertness/Sedation (OAA/S) scale (ie*,* effective sedation defined as a score of 17 or less in the 20‐point scale).^10^ As expected, it was observed that the response rates tended to decrease when the type of success criterion became more restrictive (see Table 2).

The most restrictive criterion used the OAA/S scale, specifically designed to evaluate drug‐induced sedation with benzodiazepines. The OAA/S scale was methodologically validated and evaluated for its reliability with midazolam in healthy adult subjects and has shown a high discriminatory power and a high sensitivity, using its composite score or sum score.^11^ Correlation was shown between OAA/S and other commonly used scales.^12^

The 3‐ to 5‐point scales are commonly used in current medical practice where frequent monitoring is required. Compared to the OAA/S scale, these scales can be rated more quickly (single global clinical assessment), and the rating can be more easily repeated in a short period of time (eg, every 10 minutes during a 45 minutes period) to evaluate the evolution of sedation from midazolam administration up to the start of the procedure or anesthesia induction. Rating is more subjective with 3‐ to 5‐point scales, and the OAA/S scale is more reliable for measuring sedation in clinical studies;^11^ however, only two studies were found using the OAA/S scale for the evaluation of sedation oral midazolam and fulfilling the selection criteria.^10^, ^13^

The variability of the rating scales used clearly contributes to the heterogeneity in the response rates observed in the different studies. The observed heterogeneity could be further explained by the context of sedation, and the age of the patients and the dose, but other elements of the design or the methodologies used in the different studies (eg*,* time of evaluation of sedation) and population characteristics (eg*,* their ASA status and the presence of heart disease), could also contribute to this heterogeneity.

### 3.2 Effectiveness in different contexts of sedation

**Figure 2:**
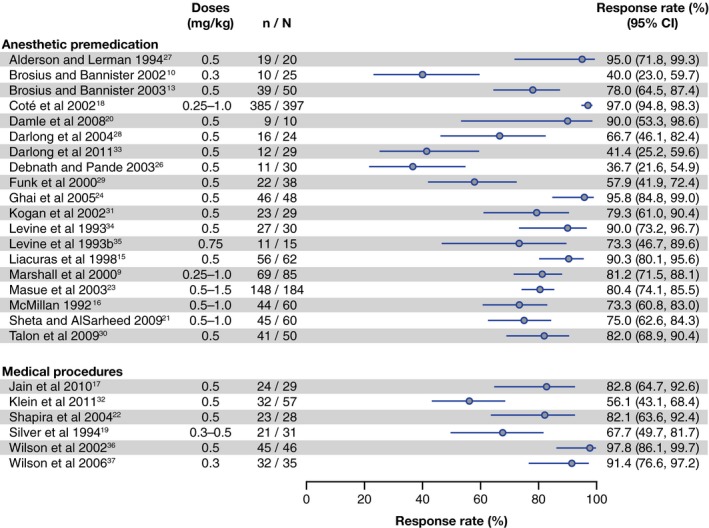
Response rates (95% CI) in the different contexts of sedation in pediatric patients receiving oral midazolam. n: number of responders, N, number of subjects

A total of 19 trials (N = 1352) reported response rates in the context of anesthetic premedication (including 3 placebo‐controlled studies) and only 6 trials (N = 258) reported response rates in the context of medical procedures (including 1 placebo‐controlled study). The studies presented high variability, with response rates [95% confidence interval] in anesthetic premedication ranging from 36.7% [21.6%, 54.9%] to 97.0% [94.8%, 98.3%] and from 56.1% [43.1%‐68.4] to 97.8% [86.1%, 99.7%] in medical procedures (Figure 2).

Although it could be argued that different sedation criteria should be used between premedication and sedation before procedures, the reality of the studies was that differences observed in sedation scales or success criteria were not associated with the context of sedation (there were studies with nonrestrictive and more restrictive success criteria both for medical procedures and for premedication before anesthesia). There was no apparent difference in terms of response rates between both contexts of sedation.

Only six of the studies fulfilled the selection criteria for sedation before procedures, and therefore, limited information could be gathered from studies performed in this indication. The effectiveness of midazolam in children undergoing procedures, such as dental treatment, has already been reported in a previous review.^14^

### 3.3 Midazolam vs placebo

**Figure 3:**
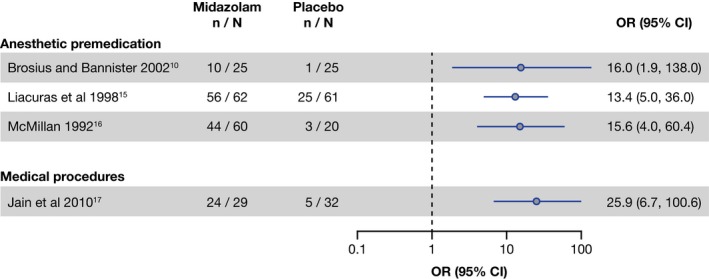
Number of responders with midazolam and placebo and OR (95% CI) calculated for the studies comparing midazolam vs placebo in the different contexts of sedation. n, number of responders, N, number of subjects

Only 4 trials compared midazolam (N = 176) to placebo (N = 138). The number of responders with midazolam and with placebo in the individual studies is shown in Figure 3. The response rates estimated ranged from 36.7% [21.6%, 58.9%] to 97.8% [86.1%, 99.7%] with oral midazolam and from 4.0% [0.6%, 23.5%] to 41.0% [29.4%, 53.6%] with placebo.

The “placebo‐effect” observed may be explained by the fact that calm children were considered as responders in most of the placebo‐controlled studies, regardless of their level of nervousness at baseline. Indeed, epidemiological data suggest that while 60% of children are anxious prior to undergoing surgery or a medical procedure, the rest may remain calm before an intervention.^4^

The OR [95% CI] ranged from 13.4 [5.0, 36.0] to 16.5 [1.9, 138.0]) for studies in premedication before anesthesia.^10^, ^15^, ^16^ The OR obtained for the single placebo‐controlled study in procedural sedation^17^ was 25.9 [6.7, 100.6] (Figure 3). Despite the observed heterogeneity, all the individual ORs estimated were clearly in favor of a statistically significantly superior response rate with midazolam when compared to placebo.

### 3.4 Comparison of midazolam doses

The dose most frequently used in the trials, irrespective of the context of sedation, was 0.5 mg/kg. Among all trials, 5 reported response rates at 0.25‐0.3 mg/kg (N = 236), 22 at 0.5 mg/kg (N = 811), 3 at 0.75 mg/kg (N = 55), 5 at 1.0 mg/kg (N = 234), and 1 at 1.5 mg/kg (N = 136). Six of the trials presented dose comparisons. In most cases, statistically significantly higher response rates were shown at doses of 1.0 or 1.5 mg/kg as compared to doses of 0.25 or 0.5 mg/kg. However, no statistically significant differences were shown between 0.25 and 0.5 mg/kg doses or between 0.75 and 1.0 mg/kg doses.

Some reports conclude that small doses of midazolam (0.25‐0.5 mg/kg) are highly effective and that little advantage is gained by increasing the dose (0.75‐1.5 mg/kg). Among them is a very well‐designed study that enrolled 397 patients and used a 5‐point scale to demonstrate satisfactory sedation at 3 midazolam doses (0.25, 0.5 and 1.0 mg/kg), where response rates of 93.2% (123/132), 97.7% (129/132), and 100% (133/133), respectively, were observed.^18^ Two out of the six studies that evaluated different doses did not observe a dose‐response trend and obtained higher response rates at low doses than at high doses.^16^, ^19^

In noncooperative children, midazolam was shown to be efficacious for dental procedures, both when used as premedication before general anesthesia (0.5‐1.0 mg/kg)^20^, ^21^ and for sedation (0.3‐0.5 mg/kg).^19^, ^22^

### 3.5 Effectiveness considering different age groups

The age of the patients included in the different studies is shown in Table 2. The age groups considered in the studies were generally not in agreement with standard ICH pediatric classification, precluding comparison between studies.

When assessed individually, the single study (N = 397) evaluating the efficacy of midazolam in different age groups indicated that, although more children in the youngest group were agitated at baseline, the response rates with midazolam according to sedation criteria were not significantly different across ages, with overall response rates of 97, 96, and 98% for children 6 months to <2 years, 2 to <6 years, and 6 to <16 years, respectively.^18^

### 3.6 Safety

Descriptive and/or quantitative safety information from the selected studies has been summarized in Table 2. The main adverse events observed were paradoxical reactions, nausea and vomiting and respiratory events. According to the available safety data from the studies, higher midazolam doses generally resulted in a higher incidence of adverse events and of cases of over‐sedation (particularly 1‐1.5 mg/kg doses). It has been shown that doses of midazolam higher than 0.5 mg/kg may be associated with increased levels of adverse events such as loss of balance and head control, dysphoria and blurred vision, hypotension, respiratory depression, dysphoric reactions, and ataxia^9^, ^16^, ^18^ and lead to a higher incidence of deep sedation.^18^, ^21^, ^23^ Cases of deep sedation with an oral dose of 0.5 mg/kg have also been reported.^24^

It was noted that some of the adverse events reported are observed when oral midazolam is used in combination with other drugs (following induction of anesthesia, or after administration of treatments used for local anesthesia, or other medications required for the respective interventions) and can therefore not be attributed to midazolam treatment exclusively.

### 3.7 Oral midazolam products used

Some of the studies were performed using commercial oral syrups of midazolam (five studies, N = 614), but the majority used liquid extemporaneous preparations made from existing parenteral formulations, generally mixed with syrups or flavorings to improve palatability (21 studies, N = 810). Unfortunately, these extemporaneous preparations suffer from a lack of standardization, particularly with regard to pH, concentration, and ingredients. In one of the studies,^24^ the parental form was administered by the oral route without preparation (N = 48). Considering the high solubility and absorption of midazolam, provided there is adequate solubilization of midazolam in the preparation, these different liquid forms cannot be considered to be different in terms of bioavailability,^25^ as the limiting factor for midazolam is first‐pass metabolism.

### 3.8 Other comparators

Certain studies used other drugs (ketamine, dexmedetomidine, pentobarbitone, hydroxyzine, nitrous oxide, alone or in combination with midazolam or with other medications) as comparators (12 studies, N = 392 treated with midazolam, N = 417 treated with other drugs), and others compared oral midazolam with alternative routes of administration (2 studies, N = 86 treated with midazolam, N = 194 treated by other routes of administration), as summarized in Table 2.

Among the studies reviewed, some compared oral midazolam 0.5 mg/kg to oral ketamine 5‐6 mg/kg and demonstrated that both presented similar efficacy.^26^, ^27^, ^28^, ^29^ However, ketamine is characterized by undesirable effects, such as excessive salivation, emesis, vertigo, and hallucinations,^28^, ^29^ and midazolam has been reported to afford shorter recovery times than ketamine.^20^, ^26^, ^27^

One of the studies indicated that midazolam 0.5 mg/kg and intranasal administration of dexmedetomidine 2 µg/kg were comparable in terms of response rates, although intranasal dexmedetomidine could be more useful when the intervention requires sleep induction.^30^

When compared to other alternative noninvasive routes, midazolam (0.5 mg/kg) administered orally produced equivalent response rates as rectal (0.5 mg/kg), intranasal (0.3 mg/kg), and sublingual (0.3 mg/kg) midazolam. Intranasal presented the most rapid onset of action but was less well‐tolerated.^31^, ^32^

Most of these alternative medications are used off‐label in children.

## 4 CONCLUSION

Oral midazolam is an efficacious medication with an adequate safety profile for use in minimal or moderate sedation in children from 4 months to 18 years old at doses from 0.25 to 1.5 mg/kg. The incidence of adverse events, such as paradoxical reactions and respiratory events, and the risk of over‐sedation increase with increasing doses. Oral midazolam compares well with other alternative noninvasive medications. The need to use validated scales for the evaluation of sedation in future studies is highlighted.

## CONFLICT OF INTEREST

MA Manso, C. Guittet and LA Granier are employees of Advicenne and hold stock options or shares in the company.
